# *Cftr* deletion in mouse epithelial and immune cells differentially influence the intestinal microbiota

**DOI:** 10.1038/s42003-022-04101-5

**Published:** 2022-10-26

**Authors:** Callie E. Scull, Meng Luo, Scott Jennings, Christopher M. Taylor, Guoshun Wang

**Affiliations:** Department of Microbiology, Immunology and Parasitology, Louisiana State University Health Sciences Center, New Orleans, LA USA

**Keywords:** Experimental models of disease, Gastrointestinal models

## Abstract

Cystic fibrosis (CF) is a life-threatening genetic disorder, caused by mutations in the CF transmembrane-conductance regulator gene (*cftr*) that encodes CFTR, a cAMP-activated chloride and bicarbonate channel. Clinically, CF lung disease dominates the adult patient population. However, its gastrointestinal illness claims the early morbidity and mortality, manifesting as intestinal dysbiosis, inflammation and obstruction. As CF is widely accepted as a disease of epithelial dysfunction, it is unknown whether CFTR loss-of-function in immune cells contributes to these clinical outcomes. Using *cftr* genetic knockout and bone marrow transplantation mouse models, we performed 16S rRNA gene sequencing of the intestinal microbes. Here we show that *cftr* deletion in both epithelial and immune cells collectively influence the intestinal microbiota. However, the immune defect is a major factor determining the dysbiosis in the small intestine, while the epithelial defect largely influences that in the large intestine. This finding revises the current concept by suggesting that CF epithelial defect and immune defect play differential roles in CF intestinal disease.

## Abstract (web summary)

The cystic fibrosis transmembrane-conductance regulator (*cftr*) gene function in epithelial and immune cells affect gut microbiome in mice differently.

## Introduction

Cystic fibrosis (CF), one of the most common and fatal autosomal recessive genetic disorders, affecting 1/~3000 live births in the United States and at least 70,000 patients worldwide^1^. The responsible gene encodes CF Transmembrane-conductance Regulator (CFTR), a cAMP-activated chloride and bicarbonate channel^2–4^. Clinically, CF targets multiple organ systems, including the lung, intestines, liver, pancreas, and reproductive system^5,6^. While the pulmonary disease dominates the adult patient population, the gastrointestinal disease that begins in utero causes early morbidity and mortality. Meconium ileus presents in up to 20% of infants with CF^7^. Despite interventions, intestinal problems, such as constipation and distal intestinal obstruction syndrome, persist throughout life^8,9^.

The innermost part of intestines is lined by an epithelium that consists of enterocytes, goblet cells, enteroendocrine cells, Paneth cells, and others. Enterocytes are the predominant cell type, occupying ~90% of the epithelium, followed by goblet cells (~8%) and enteroendocrine cells (~1%)^10^. Although Paneth cells have no detectable CFTR^11^, all the major epithelial cells have been shown to express this channel^12–14^. CFTR mediates the secretion of chloride across the epithelial layer, and plays a critical role in regulating salt and water flux into the intestinal lumen to maintain fluidity of the luminal contents^15^. CFTR also conducts bicarbonate, which is important to neutralize the acidic bile leaving the stomach^16^. A dysregulated CFTR has been linked to acidification of intestinal lumen pH^17^, small intestinal bacterial overgrowth^18,19^, large intestinal dysbiosis, and distal intestinal obstruction syndrome^20,21^. As all these pathological changes occur in the intestinal lumen, it is widely believed that CFTR defect in the epithelial cells is primarily responsible for the intestinal disease. However, emerging evidence suggests that host immune system directly affects commensal bacteria, crucial to intestinal health^22–24^. CFTR dysfunction in CF humans and mice is found to actively select the gut microbiome^25–29^, and causes heightened inflammation with increased numbers of inflammatory cells in the intestines^30,31^. Furthermore, extensive research demonstrates that CFTR loss-of-function in immune cells, including neutrophils^32,33^, monocytes^34,35^, and macrophages^36^, compromises their immune functions^34,36–42^. Despite these functional impairments, it is not determined whether the CF immune defect is directly involved in shaping the intestinal microbiota.

In this report, we hypothesized that both CF epithelial defect and immune defect jointly influence the intestinal microbiota, thus contributing to the disease pathogenesis. To test this hypothesis, we interrogated the intestinal microbiomes of WT mice and various types of CF mice with CFTR loss-of-function in either whole body or different innate immune cells. We also investigated the intestinal microbiomes of WT and CF mice with immune replacement via bone marrow transplantation (BMT). Data demonstrate that the epithelial defect and the immune defect in CF affect the intestinal microbiota differentially.

## Results

### Intestinal microbiome profiling of WT and CF mice

**Fig. 1:**
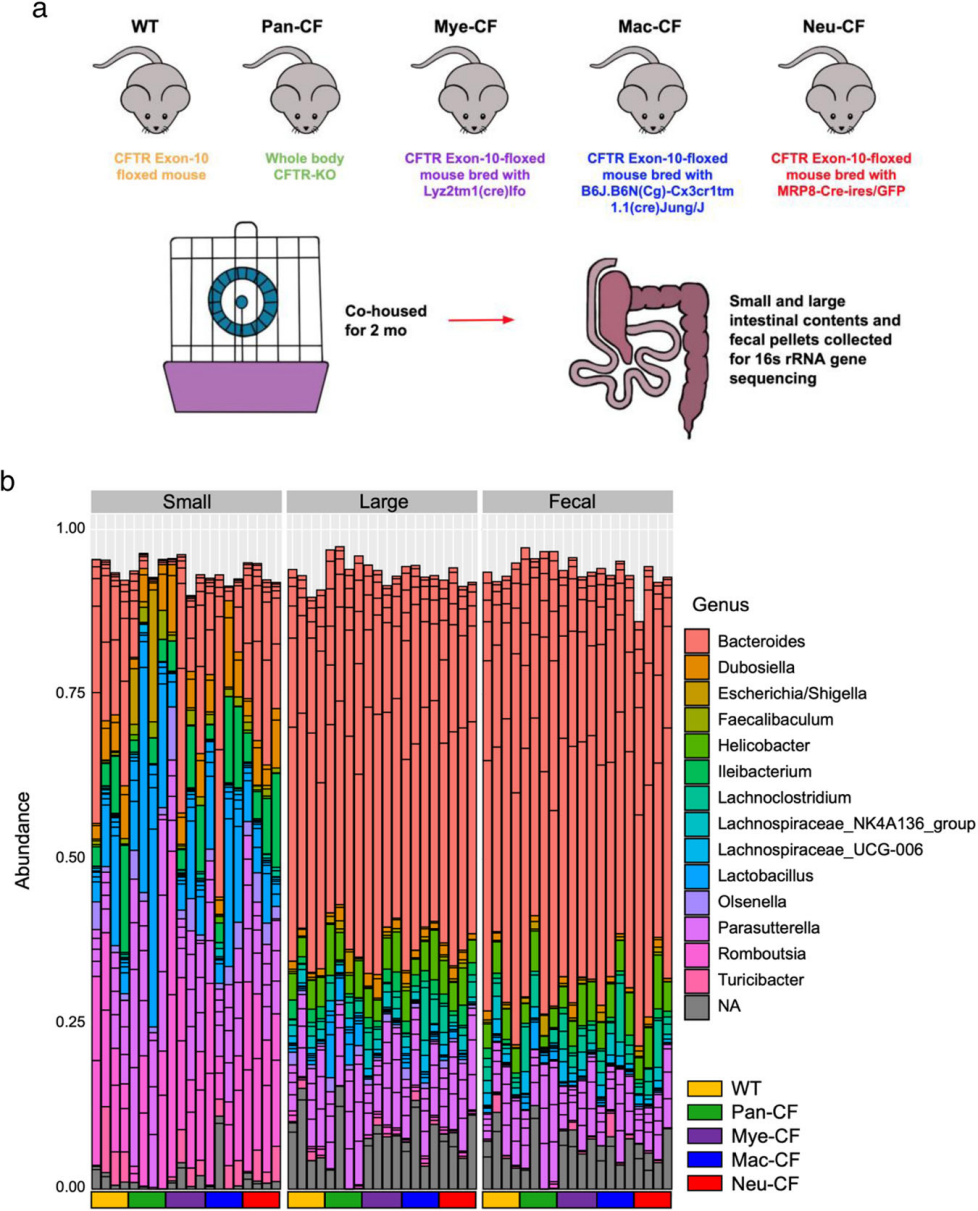
Experimental design and top bacterial taxa of gut microbiota from multi-genotype co-housing mice. **a** Schematic of multi-genotype co-housing. Five genotypes of mice (WT, Pan-CF, Mye-CF, Mac-CF and Neu-CF) were co-housed for 2 months for intestinal microbiota analyses. **b** Taxonomic data showing the relative frequency of bacterial taxa of the top 15 bacterial taxa from all co-house mice. Samples are grouped by genotype and by location (*n* = 4).

Previous studies have shown that CF humans^29^, mice^25^, and rabbits^43^ have an altered intestinal microbiome when compared to their control cohorts. However, these CF subjects had ubiquitous CFTR loss-of-function in their entire body. Thus, it is impossible to determine if the microbiome alteration was induced by the intestinal epithelial defect, the immune defect, or a combination of both. To address this issue, we had created three *cftr* knockout mouse models: Myeloid CF (Mye-CF), Macrophage CF (Mac-CF), and Neutrophil CF (Neu-CF), as described in Materials and Methods. These lines of *cftr*-deletion mice represented different scales of immune defect. Pan-CF had whole-body *cftr* deletion, the most severe form of defect. Mye-CF had neutrophil, monocyte and macrophage *cftr* deletions, the second severe form of defect. Mac-CF had *cftr* deletion only in macrophages, and Neu-CF only in neutrophils, each representing the least form of defect. Four cages of age- and sex-matched animals were established with each cage containing a WT, a Pan-CF, a Mye-CF, a Mac-CF, and a Neu-CF mouse (Fig. 1a). After co-housing for two months, fecal pellets from each animal were harvested sterilely. Then, the animals were euthanized, and the contents from their small and large intestines were separately collected. Tissues from these intestines were also preserved for histology. The obtained feces and intestinal contents were extracted for microbial DNAs and subjected to PCR amplification of the variable region of the 16S rRNA gene, followed by sequencing for microbiome profiling. A taxonomic bar graph for each sample of the top 15 bacterial taxa, inferred from the total microbiomes of all samples, was compiled to show the microbial landscapes in the intestines and feces of all animals (Fig. 1b). The taxonomic graph of the data sorted by cage is also provided (Supplementary Fig. 1 and Supplementary Data 1).

### CFTR loss of function in different tissues influences alpha and beta diversities of the intestinal microbiomes differently

**Fig. 2:**
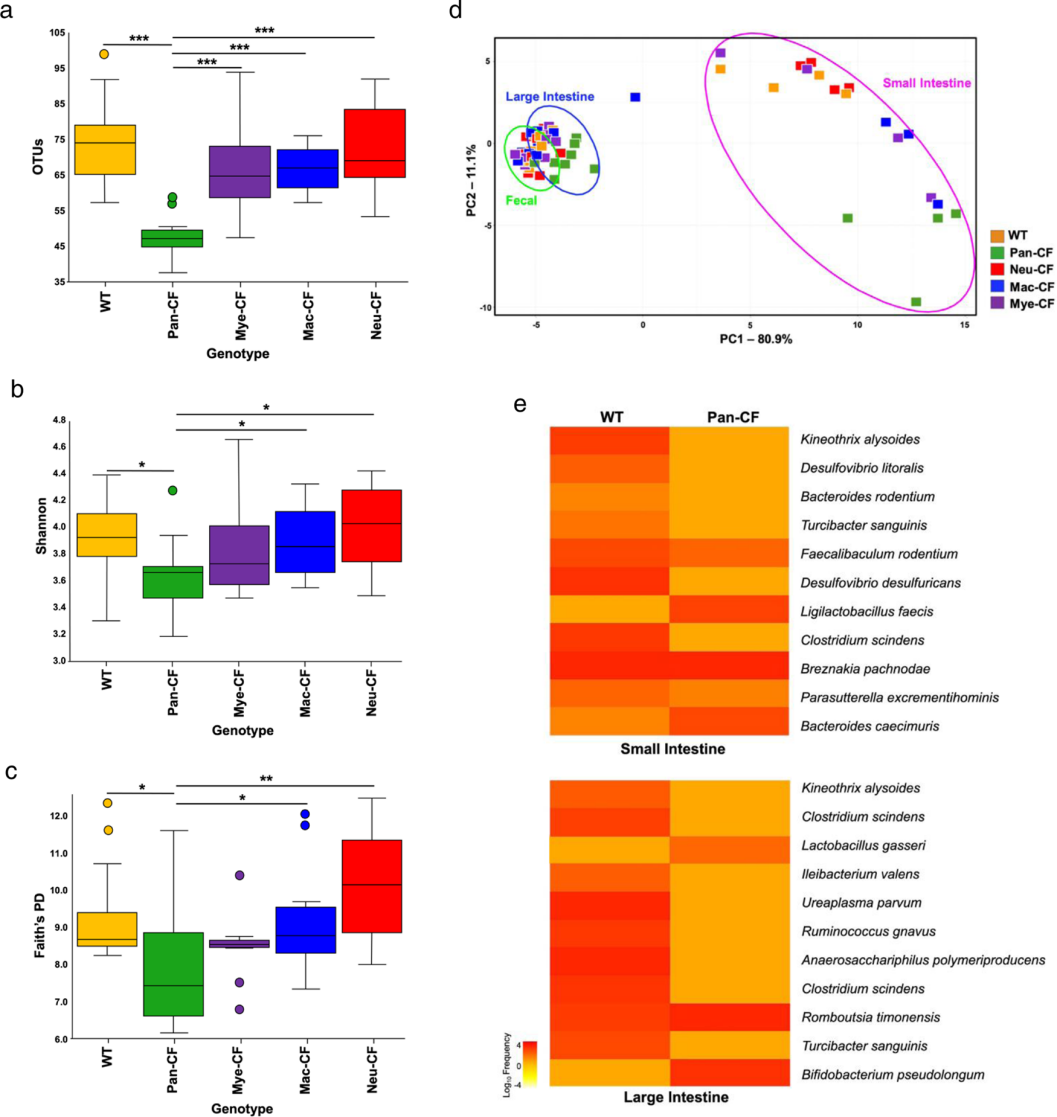
Differences in alpha and beta diversities in the gut microbiota from different genotypes of CF mice. **a** Observed OTUs were used to measure species abundance within a sample. The Pan-CF mice have a significantly reduced number of OTUs as compared to WT, Mac-CF, Neu-CF, and Mye-CF. Data were obtained from Qiime2 by Kruskal–Wallis (pairwise) comparison (***p < 0.001; *n* = 12 from four animals per genotype and three sampling locations per animal). **b** Shannon Index to measure population evenness. Pan-CF mice have a significantly reduced Shannon index when compared to WT, Mac-CF, and Neu-CF (*n* = 12). Data were obtained from Qiime2 by Kruskal–Wallis (pairwise) comparison (*p < 0.05; *n* = 12 from four animals per genotype and three sampling locations per animal). **c** Phylogenetic diversity judged by Faith’s phylogenetic diversity (Faith’s PD). The Pan-CF mice have a significantly reduced phylogenetic distance as compared to WT, Mac-CF, and Neu-CF. Data were obtained from Qiime2 by Kruskal–Wallis (pairwise) comparison (*p < 0.05 or **p < 0.01; *n* = 12 from four animals per genotype and three sampling locations per animal). **d** Bray–Curtis PCA plot to show similarity and dissimilarity of the gut microbiomes from the five genotypes at all three sampling locations. Small intestinal samples are more dissimilar, while the large intestine and fecal samples congregate (*n* = 4 for each genotype). **e** Heatmaps to compare significant differences in abundance of microbial taxa between WT vs. Pan-CF in the small and large intestine. Significance determined using FDR BH corrected *p* values (*p* < 0.05; *q* < 0.1 *n* = 4). **a**–**c** Box plots are shown as interquartile (box), median (horizontal line), minimum and maximum values (error bars).

To compare the intestinal microbiomes, we first performed alpha diversity analyses to measure variables within each sample (community), such as richness (quantity of species), evenness (proportion of species), and diversity (total number and relative abundance (RA) of species)^44,45^. Microbial richness was gauged by operational taxonomic units (OTUs) based on sequence similarities, microbial evenness by Shannon index determined by RA of the taxa present^44,46^, and microbial diversity by phylogenetic diversity (PD or Faith’s PD) defined by microbial relatedness^47,48^. Data demonstrate that Pan-CF mice had significantly reduced OTUs as compared to WT, Mye-CF, Mac-CF and Neu-CF mice (Fig. 2a and Supplementary Data 2). Furthermore, Pan-CF mice had reduced bacterial evenness (Shannon index) (Fig. 2b and Supplementary Data 3) and reduced PD (Fig. 2c and Supplementary Data 4), as compared to WT, Mac-CF and Neu-CF mice. However, Mye-CF and Pan-CF had no differences in Shannon index (Fig. 2b) and in Faith’s PD (Fig. 2c). Thus, Pan-CF mice had the most drastic changes in intestinal microbiota, and the combined CFTR loss of function in neutrophils, monocytes and macrophages in Mye-CF mice influenced the intestinal microbiota in a way similar to ubiquitous CFTR loss of function. However, *cftr* deletion in macrophages or neutrophils alone had little to no influence on the microbiota as was closer to WT mice. These data suggest that the innate immune defect contributes to the altered CF intestinal microbiota.

While alpha diversity focuses on microbial variations within a microbial community (sample), beta diversity quantifies (dis-)similarities between communities (samples). Microbiome similarity or dissimilarity can be measured by Bray–Curtis Beta diversity metrics^49^. When visualized graphically, samples plotted close to one another mean that their microbiomes share more similarities; while samples plotted far apart from one another typically have more unrelated microbiomes.

From the principle component analysis (PCA) plot, we can evaluate how similar or dissimilar the intestinal microbiomes of all mouse genotypes were related to one another (Fig. 2d). Individual PCA plots by intestinal location are also presented (Supplementary Fig. 2). It is obvious that in the small intestine Pan-CF mice were most dissimilar among the five genotypes, while Neu-CF and WT mice were most similar. Intriguingly, the loss of CFTR in Mye-CF and Mac-CF mice resulted in the most microbial diversity in this segment of intestine. In contrast, the large intestine and fecal samples congregated, but Pan-CF mice were still most dissimilar. As the small intestine is the site most susceptible to pathology and obstruction in CF, our data suggest that the host innate immunity plays a direct role in shaping the diverse mouse microbiomes at this location.

### Different scales of CFTR loss of function alters the abundances of intestinal bacterial taxa differently

We then assessed the composition of the bacterial communities within the intestines to determine how the loss of CFTR in Pan-CF and immune-specific CF mice influenced the gut microbiome compared to WT mice. Data show that Pan-CF mice had 22 bacterial taxa with significant difference in abundance, as compared to WT mice (Supplementary Table 1). Out of the 22 taxa, 11 were found in the small intestine with 3 increased and 8 decreased (Fig. 2e). Notably, the 3 increased taxa were *Faecalibaculum rodentium, Ligilactobacillus faecis*, and *Parasutterella excrementihominis*. The other 11 taxa were found in the large intestine with 2 increased and 9 decreased. The 2 increased ones were *Lactobacillus gasseri* and *Bifidobacterium pseudolongum* (Fig. 2e). Furthermore, Mye-CF mice had 1 taxon with significant difference in abundance as compared to WT mice. *Desulfovibrio* was significantly less in the Mye-CF small intestine (Supplementary Table 2). Intriguingly, Neu-CF had the similar *Desulfovibrio* difference in the small intestine (Supplementary Table 3). Altogether, these data suggest that ubiquitous loss of CFTR results in greater changes in overall intestinal microbial taxa, and the innate immune defect can affect the taxa but to a lesser extent.

### CF innate immune defect leads to significant increase in goblet cell density

**Fig. 3:**
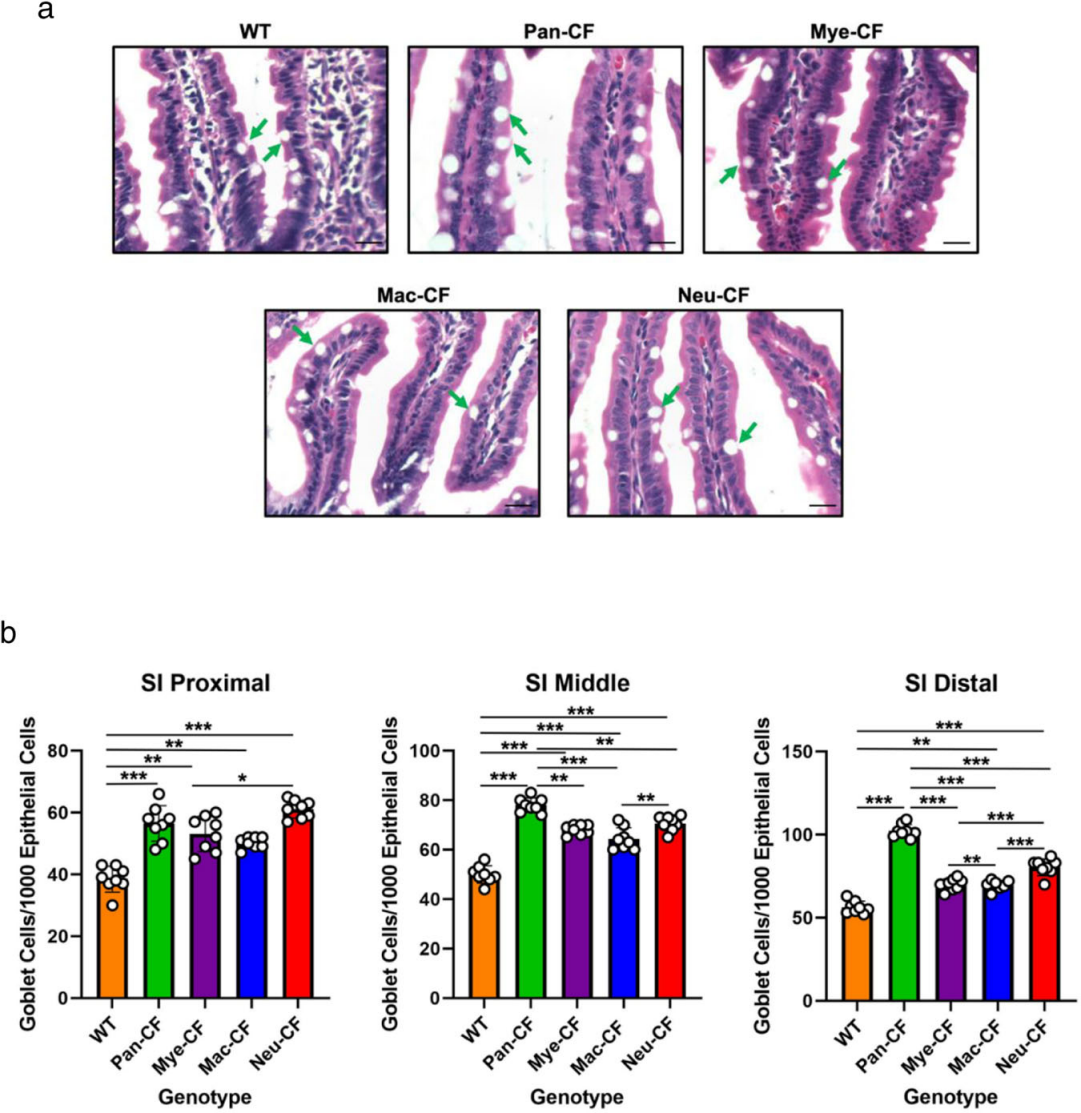
Loss of CFTR in different tissues affects goblet cell intensity in the small intestine at different levels. Tissues from the proximal, middle, and distal regions of the small intestines were processed for paraffin-embedment. Slides were stained with Hematoxylin-Eosin (HE) dyes for histological examination and goblet cell counting. **a** Representative villi from the small intestine of each genotype showing goblet cells (arrows). Scale bars correspond to 30 µm. **b** Goblet cells were counted out of 1000 enterocytes from randomly selected views of villi. Eight tissue pieces were counted from each genotype (*n* = 8). Statistic differences were determined by two-tailed unpaired Student’s *t* test. Data are shown as mean ± SD (***p < 0.001, **p < 0.01, and *p < 0.05).

Goblet cells play an important role in intestinal function by secreting mucins to form a mucus layer to coat the gastrointestinal tract^50^. Goblet cell hypertrophy and hyperplasia phenotypically reflect the severity of CF intestinal disease. More importantly, the microbiome was reported to influence goblet cell numbers in the intestines^51,52^. Thus, we predicted that the altered intestinal microbiota in the different CF mice should affect goblet cell density. Because small intestine is the most obstructive site in CF, we thus focused our goblet cell examination on this location. The proximal, middle, and distal regions of the small intestine were processed for histological examination, and goblet cells were counted against the enterocytes in randomly selected villi (Fig. 3a). Quantitative data of the five genotypes demonstrate that across all three regions of the small intestine, Pan-CF, Mye-CF, Mac-CF and Neu-CF all had a significantly higher goblet cell density than did WT mice (Fig. 3b and Supplementary Data 5). Furthermore, in the middle and distal regions, Pan-CF mice had a significantly higher goblet cell density than Mye-CF, Mac-CF and Neu-CF mice. Therefore, loss of CFTR in the innate immune cells leads to an increased density of goblet cells in the small intestine, and additional epithelial defect exacerbates this CF phenotype.

### Intestinal microbiome profiling of BMT mice

**Fig. 4:**
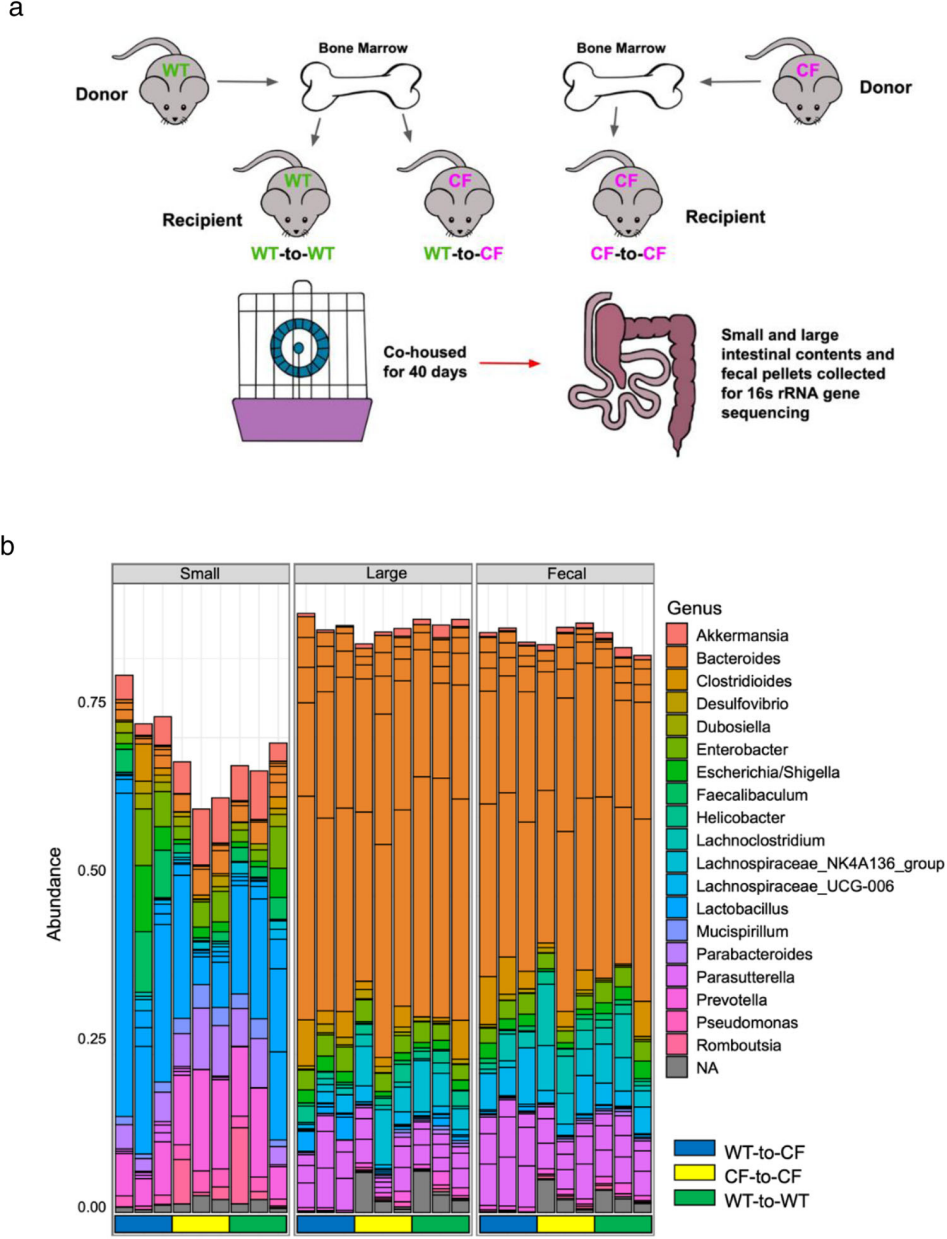
Experimental design for bone marrow reconstitution and taxonomic overview of gut microbiota. **a** Schematic of donor and recipient matching for bone marrow transplantation (BMT) and post-BMT co-housing. **b** Taxonomic data showing the relative frequency of each bacterial taxon from top 20 bacterial taxa in all BMT mice by Genotype. Data are grouped by donor-to-recipient and by intestinal location.

To confirm the above results from the genetic *cftr*-deletion mice, we performed BMT between WT and Pan-CF sibling mice, as diagrammed (Fig. 4a). This design would diminish any potential confounding variables from genetic backgrounds. Three groups of animals were established: Group #1—WT mice transplanted with WT marrow cells (WT-to-WT), Group #2—CF mice transplanted with WT marrow cells (WT-to-CF), and Group #3—CF mice transplanted with CF marrow cells (CF-to-CF). As a result, the WT-to-WT mice had CFTR expression in all epithelial and immune cells, the WT-to-CF mice had CFTR expression only in immune cells, and the CF-to-CF mice had no CFTR expression in any cells. Three co-housing cages were set with each cage containing one animal from each group. Intestinal and fecal microbiomes were similarly profiled. To assess how CF immune defect contributes to changes in the microbiome, we first compared Group #2 with Group# 3 to appreciate the immune influence on the microbiome. Then, we compared Group #1 and Group #2 to determine the epithelial influence on the microbiome. Taxonomic bar graph of each sample of the top 20 bacterial taxa, inferred from the total microbiome of all samples, was compiled to show the overview of the microbiomes in the intestines and feces of all animals (Fig. 4b). The taxonomic graph of the data sorted by cage is also presented in Supplementary Data (Supplementary Fig. 3 and Supplementary Data 6).

### CF epithelial defect and immune defect influence intestinal microbiota differentially in BMT mice

**Fig. 5:**
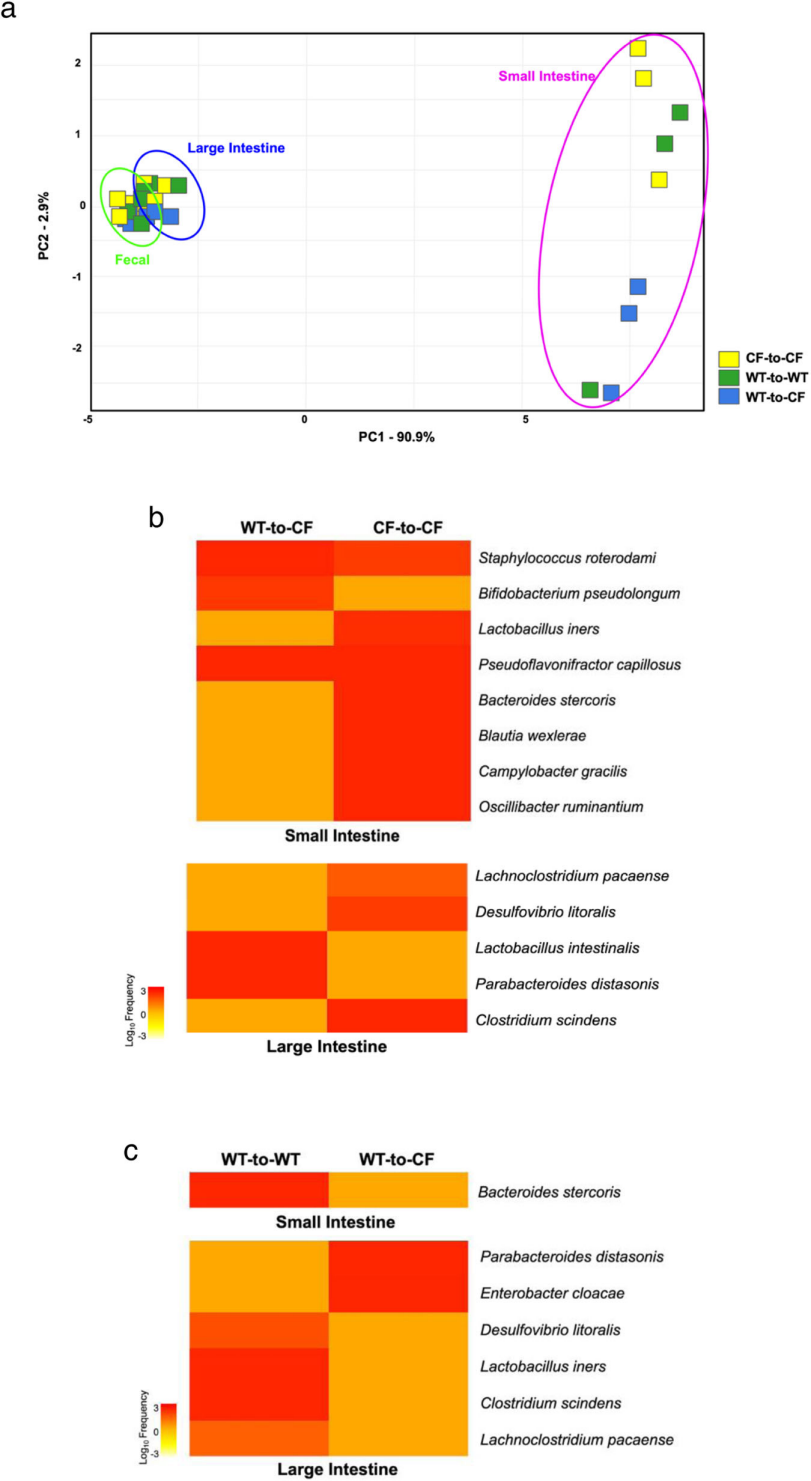
Influences of CFTR loss-of-function in epithelial cells and in immune cells on gut microbiota. **a** Dissimilarity of gut microbiota by Bray–Curtis PCA plot (*n* = 3). **b** Heatmaps to demonstrate significant differences in abundance of microbial taxa between WT-to-CF and CF-to-CF in the small and large intestines (large intestine *p* < 0.05 and *q* ≤ 0.1, *n* = 3). **c** Heatmaps to demonstrate significant differences in abundance of microbial taxa WT-to-WT and WT-to-CF in the small and large intestines. Significance determined using FDR BH corrected *p* values (*p* < 0.05 and *q* ≤ 0.1, *n* = 3).

To judge inter-group microbiome similarity or dissimilarity in the BMT mice, we performed beta diversity analysis on the three groups. Results show that the most substantial microbial changes occurred in the small intestine (Fig. 5a). PCA plots by locations are also provided (Supplementary Fig. 4). The microbiomes of CF-to-CF mice were close to each other, from which the microbiomes of WT-to-CF mice were distant, indicating that alteration of the immune system resulted in the microbiome change in this location.

To specifically address how the immune system alters the gut taxa in these mice, we analyzed differentially abundant taxa in the small and large intestines of the BMT mice. The heatmaps (Fig. 5b) show that WT bone marrow affected the abundance of intestinal bacterial taxa in CF recipient mice. In the small intestine, altering the immune system (WT-to-CF vs. CF-to-CF) impacted 8 taxa significantly, with 3 increased in WT-to-CF mice as compared to CF-to-CF (Fig. 5b). In the large intestine, the same immune alteration significantly affected 5 taxa. These bacterial taxa with different RA are listed (Supplementary Table 4). In contrast, the epithelial defect (WT-to-WT vs WT-to-CF) influenced 1 taxon in the small intestine, and 6 taxa in the large intestine (Fig. 5c), which are also listed (Supplementary Table 5). From these data, we conclude that the small intestinal microbiota was more sensitive to host immune modulation than the large intestinal microbiota. Hence, CF immune defect is a major contributor to small intestine dysbiosis seen in CF.

### Predictive functional analysis using PICRUSt2

Different microbes have different signatures of gene functionality, which are ultimately responsible for different microbial behaviors. To investigate how the host selection of intestinal microbiota in CF imprints the bacterial functionality, Phylogenetic Investigation of Communities by Reconstruction of Unobserved States (PICRUSt2) was used to predict microbial functional profiles from the metagenomics data of our five-genotype co-housing and BMT co-housing experiments. The obtained 16S rRNA gene sequencing data were compared to obtain the RA of predicted Kyoto Encyclopedia of Genes and Genomes (KEGG) pathways or KEGG orthology (KeO) features among the five-genotype mice as well as among the BMT mice (Supplementary Data 7 and 8).

**Fig. 6:**
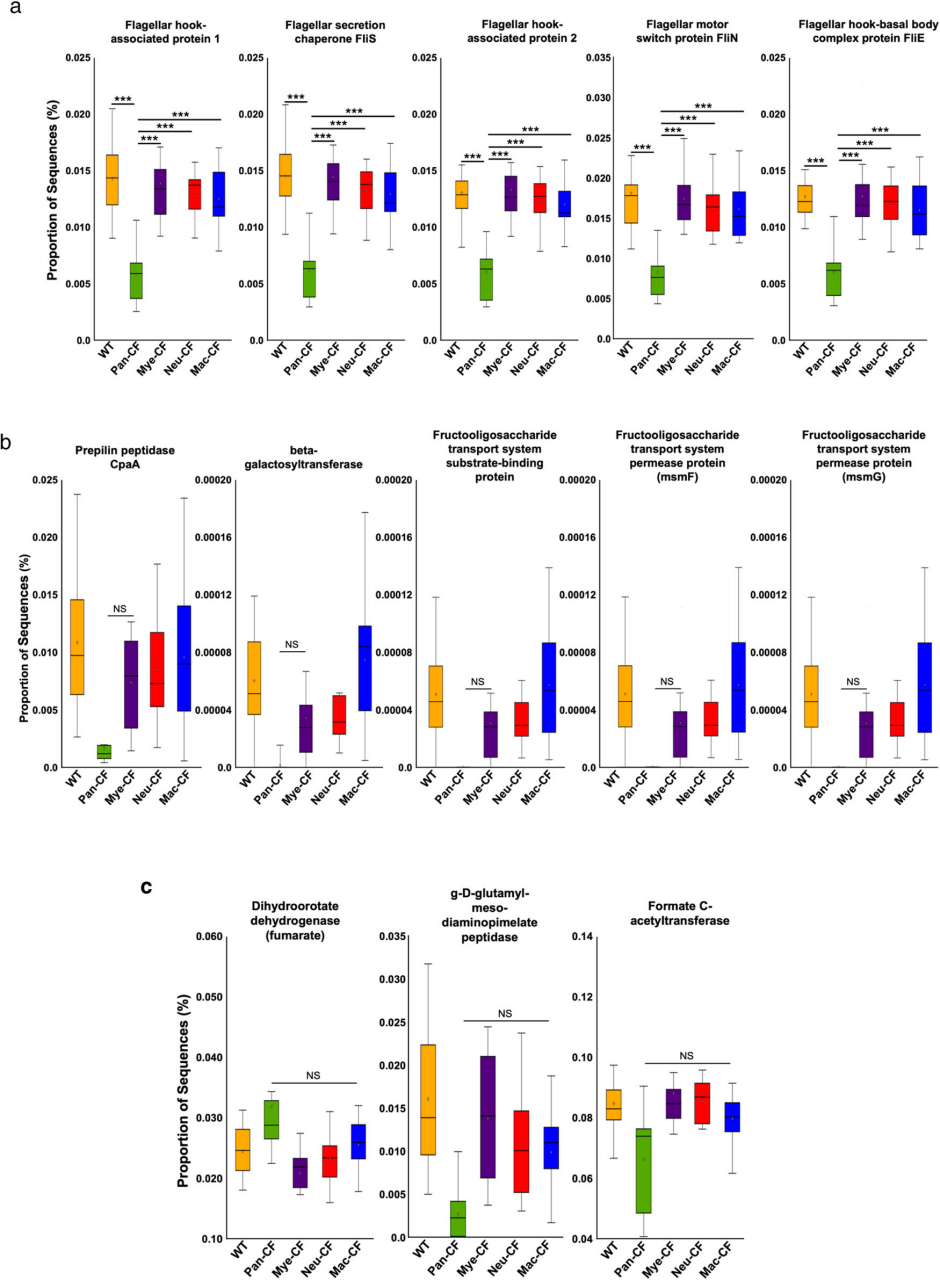
Comparison of KEGG Ortholog predictive functionality via PICRUSt2. **a** Top 5 significant differences in PICRUSt2-predicted KEGG pathways between multi-genotype co-housed animals (***p < 0.001). **b** Top 5 PICRUSt2-predicted KeO pathways with no difference between Pan-CF and Mye-CF. **c** Top 3 PICRUSt2-predicted KeO pathways with no difference between Pan-CF and Mac-CF, suggesting the importance of the respective innate immunity in modulating these aspects of microbial functionality. Multiple-group comparisons were analyzed using ANOVA and Tukey-Kramer (*n* = 12 from four animals per genotype and three sampling locations per animal) (*p* < 0.05 for the shown KEGG features) (***p < 0.001). **a**, **b** Box plots are shown as interquartile (box), median (horizontal line), minimum and maximum values (error bars).

Data from the five-genotype co-housing experiment revealed a total of 81 significant KeO features from multiple comparisons using ANOVA, Tukey-Kramer, and Benjamini-Hochberg false discovery rate (FDR) (Supplementary Data 9). The top 5 were associated with flagellar functions (flagellar hook-associated proteins 1 and 2, flagellar motor switch protein, flagellar secretion chaperone, and flagellar hook-basal body complex protein), which were significantly decreased in Pan-CF mice as compared to Mye-CF, Mac-CF, Neu-CF and WT mice (Fig. 6a), implying that the ubiquitous CFTR loss of function selects for bacteria with weakened flagellar function.

We also focused on five specific KeO features that did not show any significant differences between Pan-CF and Mye-CF. As these CF mice shared the same innate immune defect, we thus reasoned that these non-altered specific KeO features would be likely associated with the innate immune defect. As displayed (Fig. 6b), these features were mostly related to molecular modification, such as β-1,6-Galactosyltransferase, a key enzyme that adds galactose residues to galactan chains^53^, prepilin peptidases that cleave prepilin for pili formation^54^, and carbohydrates/glycans and glycosyltransferases that decorate proteins and lipids^55^. Abnormal modifications of carbohydrates, proteins and lipids can lead to unregulated activation of the immune system and exuberate inflammatory responses^56^. Moreover, we noted that there were 3 KeO features that did not show any difference between Pan-CF and Mac-CF (Fig. 6c). These features involve g-D-glutamyl-meso-diaminopimelate peptidase, a metalloproteinase; dihydroorotate dehydrogenase (DHODH), an enzyme for de novo pyrimidine biosynthesis^57^; and formate c-acetyltransferase, an enzyme in fermentation of pyruvate^58^.

**Fig. 7:**
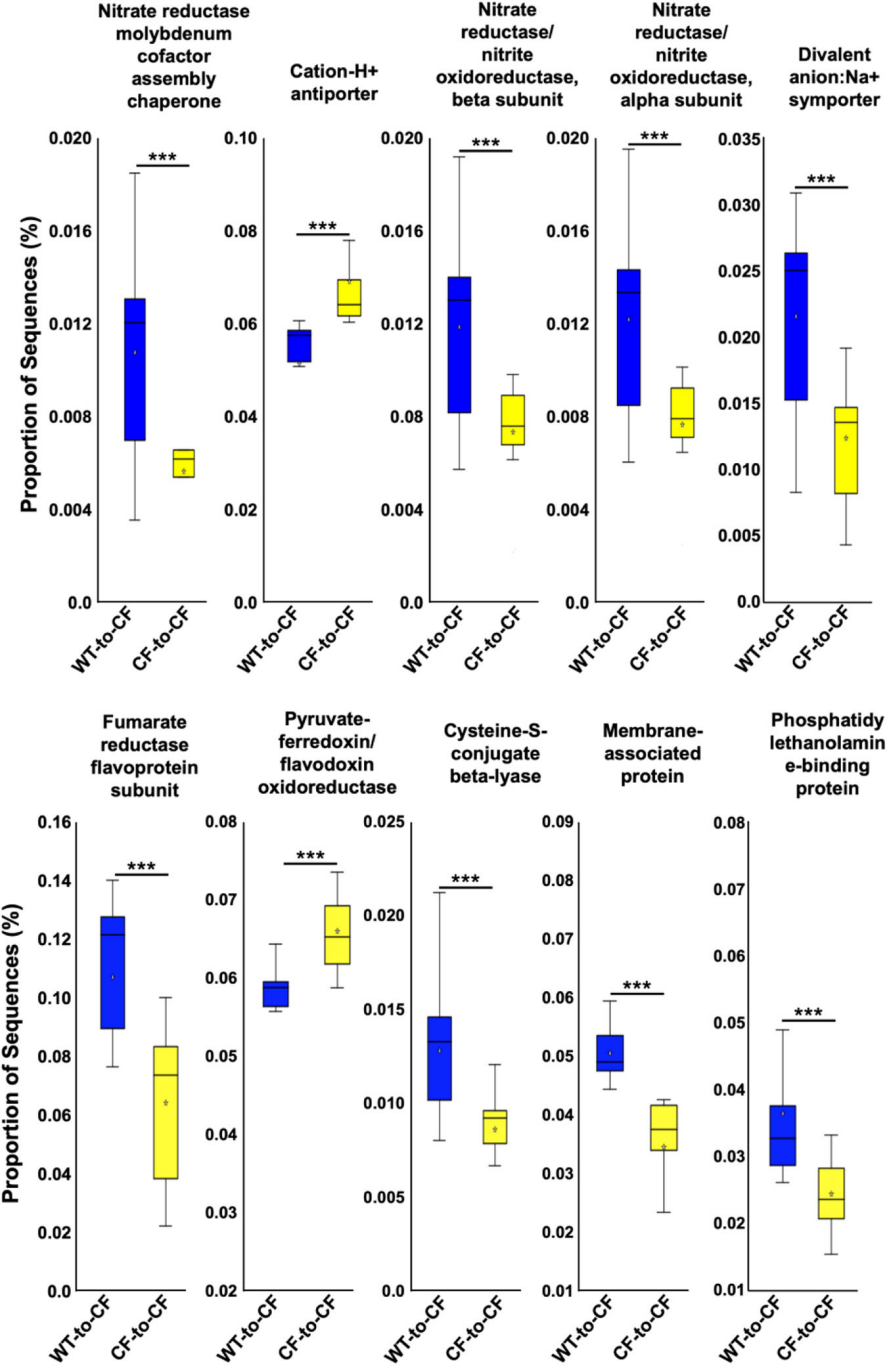
Immune and epithelial comparison of KEGG ortholog predictive functionality via PICRUSt2. **a** Top 10 significant differences in KEGG pathways between WT-to-CF vs. CF-to-CF microbiota. Two-group comparisons were done using Welch’s *t*-test (*p* < 0.05 for the shown KEGG features) (***p < 0.001). Box plots are shown as interquartile (box), median (horizontal line), minimum and maximum values (error bars).

We next analyzed the predicted functions of the microbiomes across the BMT mice. Comparing WT-to-CF with CF-to-CF mice, which differed only in the immune system, revealed 250 significant KeO features (Supplementary Data 10), with the top 10 involved in metabolism, cell signaling and environmental information processing. Eight of these ten features were upregulated in WT-to-CF gut microbiota, suggesting that loss of CFTR function in the immune system diminishes the bacteria with such functions (Fig. 7). Specifically, nitrate reductase/nitrate oxidoreductase alpha and beta subunits, Nitrate reductase molybdenum cofactor assembly chaperone NarJ/NarW, divalent anion-Na^+^ symportor, fumarate reductase flavoprotein subunit, cysteine-S-conjugate beta-lyase, membrane-associated protein, and phosphatidylethanolamine-binding protein were significantly increased in the WT-to-CF gut microbiota as compared to the CF-to-CF gut microbiota. In the opposite direction, cation-H^+^ antiporter, and pyruvate-ferredoxin/flavodoxin oxidoreductase were significantly decreased in the WT-to-CF microbiota. These data have validated the finding from the five-genotype co-housing experiment, indicating that the host immune system has a unique role in modulating the gut microbiota functionality.

## Discussion

CF patients have increased risk of intestinal inflammatory disorders. Video-equipped capsule endoscopy reveals obvious intestinal morphological abnormalities including edema, erythema, mucosal breaks, and ulcerations in the jejunum and ileum in ~60% of CF patients. Fecal neutrophil marker proteins are also significantly elevated^59^. Strikingly, CF patients have an increased prevalence of Crohn’s disease, up to 12.5-fold greater than in the general population^60^. Celiac disease, a destructive autoimmune condition of the small intestinal mucosa, is also more prevalent in CF^61,62^. While all these conditions are highly related to host immunity, it is not clearly defined whether the CF-inflicted immune system participates in shaping intestinal microbiota. Our current study suggests that CF immune defect contributes to gut dysbiosis. This finding refines the current theory that attributes CF intestinal disease solely to the epithelial defect.

Our data demonstrated that the scope of CFTR loss of function in tissues correlates with the degree of alteration of bacterial taxa in mice, reflected by the finding that multiple-tissue CFTR loss of function in Pan-CF mice influenced the microbiota most. When compared to WT mice, Pan-CF had 22 significantly different bacterial taxa, Mye-CF had 1, Neu-CF had 1 and Mac-CF had none, indicating that the innate immune defect influences taxa of the gut microbiota, albeit less significantly than the whole-body defect. These data indicate that the combination of the epithelial defect and the innate immune defect had the maximal effect on gut microbiota. Of note specifically, *Bifidobacterium* and *Lactobacillus* were significantly higher in population within the microbiome of Pan-CF mice, which may result from their superior ability to survive in the CF-acidified intestinal environment. *Clostridiales* and other taxa of the Firmicutes phylum were decreased in Pan-CF mice. Consistent with what was seen in humans^29^ and concordant with previous mouse studies^18,25^, ubiquitous loss of CFTR in mice (Pan-CF) caused a decrease in microbial diversity, richness, and evenness at all sample locations as compared with WT mice. Noteworthy from our current study is that Mye-CF mice were not significantly different from Pan-CF mice in their microbiome evenness and PD, indicating that the two models are the closest in gut microbiota among the five genotypes.

The CF small intestine is the most vulnerable site that suffers bacterial overgrowth and obstruction (meconium ileus)^63^. Intriguingly, both our five-genotype co-housing and BMT co-housing experiments demonstrated that CF immune defect plays a greater role in defining the microbiome in the small intestine, suggesting a primary role of the host immune system in the pathogenesis of CF disease at this location. A recent publication reported that manipulation of host immune system in a different experimental model mostly affects the small intestine microbiota^64^, supporting our conclusion. Multiple mechanisms may be involved in such microbial selection, potentially through direct action by immune cells, stimulation of epithelial release of mucus and anti-microbial agents, and/or modulation of intestine homeostasis^65^. We would like to point out that our BMT was between sibling WT and Pan-CF mice, which provided a well-controlled set of data to understand the relationship between CFTR function and gut microbiota. These mice were born to the same parents and housed together in the same litters. Thus, variations from genetic and environmental factors had been substantially minimized, if not totally eliminated. Reconstitution of the CF mice with the WT immunity led to changes in gut microbiota, especially in the small intestine. This provides a direct proof that CF host immunity is involved in shaping gut microbiota.

Microbial functionalities determined by PICRUSt2 predicted how loss of CFTR in the epithelial or immune system influenced the gut microbiota in our five-genotype co-housing experiment, and how alteration of the host immunity by BMT altered the gut microbiota. Among the five-genotype mice, CFTR loss of function in epithelia selected for microbes with reduced bacterial flagellar functions (Fig. 6a). It is known that individuals with CF have reduced peristalsis^66^, and have increased mucus accumulation^67^ in the intestinal tract. It is argued that such a gut environment diminishes the flagella-driving movement for microbes.

Among the outstanding KeO features we highlighted (Fig. 6b), the potential function of beta-glycosyltransferase in the Pan-CF and Mye-CF microbiomes was substantially reduced as compared to WT, Mac-CF and Neu-CF. This enzyme catalyzes the transfer of saccharide moieties from an activated nucleotide sugar (also known as the glycosyl donor) to a nucleophilic glycosyl acceptor molecule, the nucleophile of which can be oxygen-, carbon-, nitrogen-, or sulfur-based^68^. Glycosyl transfer can occur to carbohydrates, proteins, and lipids. Microbes without proper glycosylation decoration may have stronger inflammogenicity, which could lead to stronger inflammatory responses. Moreover, the microbes in Pan-CF and Mac-CF mice shared a feature with increased proportion of dihydroorotate dehydrogenase (DHODH), an enzyme involved in *de novo* pyrimidine biosynthesis. This may facilitate overgrowth of certain bacteria in the Pan-CF and Mac-CF mice (Fig. 6c).

From the BMT co-housing experiment, altering the immune system (WT-to-CF vs. CF-to-CF) caused alterations in not only microbial metabolism, but also in cell signaling and environmental information processing (Fig. 7). Out of the top ten KEGG features, eight were higher in proportion and two were lower in proportion after the immune replacement from CF to WT. The eight higher in proportion features were nitrate reductase/nitrate oxidoreductase alpha and beta subunits, nitrate reductase molybdenum cofactor assembly chaperone NarJ/NarW, divalent anion-Na^+^ symportor, fumarate reductase flavoprotein subunit, cysteine-S-conjugate beta-lyase, membrane-associated protein, and phosphatidylethanolamine-binding protein, all of which were related to bacterial metabolism, ion transport and signaling. The two lower in proportion features were cation-proton antiporter, and pyruvate-ferrodoxin/flavodoxin oxireductase. Notably, the cation-proton antiporter has been linked to increased virulence of bacteria, as the enhanced antiporters help bacteria cope with varying pH, salts, and osmolarity^69^, and expel antibiotics, thus reducing antibiotic efficacy^70^. The pyruvate-ferrodoxin/flavodoxin oxireductase is an important enzyme in the oxidative decarboxylation of pyruvate to acetyl-CoA and CO_2_^71^. Intriguingly, pyruvate-ferrodoxin/flavodoxin oxireductase has been linked to increased virulence in eukaryotic pathogens by enhancing cyto-adherence and subcutaneous abscess formation^72^. Although a direct link in pyruvate-ferrodoxin/flavodoxin oxireductase and bacterial virulence has not been found, this enzyme is crucial for anaerobic growth and bacterial survival^73^. Additionally, this enzyme is found in sulfate-reducing bacteria, which are prevalent in the gut of humans and mice, and most often isolated from individuals with uncreative colitis and inflammatory bowel disease^74^. Thus, CF immune defect not only influences bacterial taxa, but also bacterial functionalities within the taxa, which may exacerbate the pathology of the CF intestines by promoting bacterial virulence, enhancing survival in a stressful environment, and over-activating the immune system.

In summary, our study has provided data to suggest that both epithelial defect and immune defect in CF collectively influence the intestinal microbiota. However, each has its own unique role in modulating the microbial community. Thus, the innate immune defect, a previously unrecognized contributor, may play as critical a role as the epithelial defect in CF intestinal disease pathogenesis.

## Methods

### Animal research ethics

C57BL/6 mouse strain and its derived lines were used in this research, through which all relevant ethical regulations were complied with. The animal breeding and experimental protocols were approved by the Institutional Animal Care and Use Committee of Louisiana State University Health Sciences Center.

### Animals

Mye-CF mice were bred between CFTR-Exon-11-floxed mice^75^ and Lyz2tm1(cre)Ifo mice (The Jackson Laboratory, Bar Harbor, ME), Neu-CF mice between CFTR-Exon-11-floxed mice and MRP8-Cre-ires/GFP (The Jackson Laboratory), and Mac-CF mice between CFTR-Exon-11-floxed mice and B6J.B6N(Cg)-Cx3cr1tm1.1(cre)Jung/J (The Jackson Laboratory). The animals with homozygous CFTR-Exon-11-floxed alleles and lineage-specific Cre expression were selected for use by genotyping. Pan-CF mice were whole-body CFTR-exon-11 deletion mice, gifted by Dr. Mitchell Drumm at Case Western University, of which heterozygotes were phenotypically normal and only homozygotes developed severe intestinal disease.

### Multi-genotype mouse co-housing

Each cage co-housed 5 animals (6–11 weeks old, female) with one from each genotype (WT, Pan-CF, Mye-CF, Mac-CF and Neu-CF). These animals were maintained on Colyte electrolyte water (Polyethylene glycol 3350 (17.91 mM), NaCl (25.03 mM), KCl (9.61 mM), NaHCO_3_ (20.00 mM), and Na_2_SO_4_ (40.01 mM)), and raised together for 2 months to normalize for potential environmental influences on gut and fecal microbiota.

### Bone marrow transplantation (BMT) and BMT animal cohousing

WT and Pan-CF recipients were irradiated at a dose of 9.5 Gy from a gamma radiation source. Bone marrow cells were isolated from heterozygous (CFTR+/−) or homozygous (CFTR−/−) Pan-CF mice. After viable cell counting, isolated bone marrow cells (~1 × 10^6^ in 100 μl of cell culture media) were injected into corresponding recipients via retro-orbital injection. Mice were grouped into three experimental groups: (1) WT receiving BM from CFTR+/− mice (WT-to-WT), (2) CFTR−/− receiving BM from CFTR+/− mice (WT-to-CF), and (3) CFTR−/− mice receiving BM from CFTR−/− mice (CF-to-CF). As CFTR+/− mice were phenotypically as normal as CFTR+/+, we chose CFTR+/− mice as the WT donors for the convenience of tracing bone marrow reconstitution via genotyping. All allocated mice from each of the three groups were co-housed for 40 days on the Colyte electrolyte water.

### Intestinal and fecal sample collection

For fecal sample collection, each mouse was placed in a sterile container, and 5 to 7 pellets were harvested at one time, snap-frozen in liquid nitrogen, and stored at −80 °C. For intestinal sample collection, each mouse was euthanized by CO_2_ asphyxiation. The small intestine was excised at the pyloric sphincter and the ileocecal valve. The large intestine was excised at the junction between the cecum and the large intestine, and at the base of the colon. Each intestine was flushed with 1–3 ml sterile ice-cold PBS (Gibco). For histology, a small segment (2–3 mm) were excised from the proximal, middle, and distal region of each intestine, and fixed in 4% paraformaldehyde for further processing.

### Tissue processing for histology and goblet cell counts

Fixed in 4% paraformaldehyde, tissue samples from the proximal, medial, and distal regions of the small and large intestines were sent to the Morphology and Imaging Core at Louisiana State University Health Science Center for processing. Briefly, tissues underwent routine histological processing including paraffin embedding and downstream processes for Hematoxylin and Eosin (H&E) staining. The slides were examined and analyzed under a microscope. Goblet cells were enumerated out of 1000 cells in any chosen villi from random areas of each tissue piece (*n* = 8). Numbers were averaged and converted to percentages. Statistical significance was judged by Student’s *t* test.

### DNA extraction, PCR amplification and sequencing

DNA extraction and sequencing were performed by the Louisiana State University School of Medicine Microbial Genomics Resource Group (http://metagenomics.lsuhsc.edu/). The genomic DNA was extracted using the QIAamp DNA Stool Mini Kit (Qiagen, Valencia, CA) modified to include bead-beating and RNase A treatment. A negative control was set for checking any potential bacterial DNA existing in chemicals or involved during the DNA extraction process. Two steps of amplification were performed to prepare sequencing library using the AccuPrime Taq high fidelity DNA polymerase system (Invitrogen, Carlsbad, CA). A negative control with the control from DNA extraction and a positive control of Microbial Mock Community HM-276D (BEI Resources, Manassas, VA) were set during amplicon library preparation. 16S ribosomal DNA hypervariable region V4 was amplified using 20 ng genomic DNA and the gene-specific primers with Illumina adapters: Forward 5’-TCGTCGGCAGCGTCAGATGTGTATAAGAGACAGGTGCCAGCMGCC GCGGTAA-3’; Reverse 5’-GTCTCGTGGGCTCGGAGATGTGTATAAGAGACAG GGACTACHVGGGTWTCTAAT-3’. The PCR conditions were as follows: 95 °C for 3 min; 25 cycles of 95 °C for 30 s, 55 °C for 30 s and 72 °C for 30 s; 72 °C for 5 min; and holding at 4 °C. PCR products were purified using AMPure XP beads added as 0.85x PCR volume. The purified amplicon DNA (2 µl) from the last step was amplified 8 cycles under the same PCR condition using primers with different molecular barcodes: forward 5’-AATGATACGGCGACCACC GAGATCTACAC [i5] TCGTCGGCAGCGTC-3’; reverse 5’-CAAGCAGAAGACGGCAT ACGAGAT [i7] GTCTCGTGGGCTCGG-3’. The indexed amplicon libraries purified using AMPure XP beads and quantified using Quant-iT PicoGreen (Invitrogen) were normalized and pooled. The pooled library was quantified using KAPA Library Quantification Kit (Kapa Biosystems), diluted and denatured as the guideline of Illumina’s sequencing library preparation. 10% PhiX was added to the sequencing library as an internal control and to increase diversity of 16S RNA amplicon library. The paired-end sequencing was performed on an Illumina MiSeq (Illumina, San Diego, CA) using the 2 × 250 bp V2 sequencing kit.

### Bioinformatics

Raw FASTQ data files were imported into QIIME2 for data analysis^76,77^. The quality of sequencing reads was determined with DADA2 pipeline version 1.22.0 to detect amplicon sequence variants with truncation of reads to 240 bp to remove low quality tails of sequencing reads and trimming of 20 bp from the left of each read to remove 16S primer sequences^78^. Taxonomic assignment of each sample was performed using R version 4.1.2 and SILVAversion 1.38.0 database^79^ (Supplementary Data 11 and 12). Before differential abundance testing, data were filtered to remove low abundance amplicon sequence variants with a mean abundance of less than 1e−4. Differential abundance was analyzed using MaAsLin2 (http://huttenhower.sph.harvard.edu/maaslin). The 16S rRNA gene amplicon data were compared between genotypes. We selected differentially abundant taxa based on an FDR-adjusted p-value. Heatmaps and taxa tables based on RA were made for the significant bacterial taxa using −log10 Transformed data and using RStudio, gplots library, heatmaps.2 package (http://www.rstudio.com/). Taxonomic bar graphs indicating top taxa abundance were created in R. Data required for the Bray–Curtis dissimilarity plot were obtained from view.qiime2.org with primary analyses via RStudio. Ggbiplot library and prcomp package were used to generate the PCA plot. Alpha diversity plots for OTUs, Shannon index, and Faith’s PD were obtained from view.qiime2.org.

### Functionality analysis

Phylogenetic Investigation of Communities by Reconstruction of Unobserved States (PICRUSt2) was used to perform predictive functionality analyses. PICRUSt2 pathway tool was used by following the github tutorial script (https://github.com/picrust/picrust2/wiki/PICRUSt2-Tutorial-%28v2.3.0-beta%29). Metagenomic prediction results were visualized using STAMP software to generate the significant KEGG function tables (https://beikolab.cs.dal.ca/software/STAMP). STAMP was also used to generate the top significant KEGG functions and the highlighted KEGG functions.

### Statistics and reproducibility

Alpha-diversity statistics were obtained from QIIME2. Goblet cell histological data were tested using Prism software (version 9.3.0; GraphPad Software, La Jolla, CA). PICRUSt2 data were visualized using STAMP, multiple-group comparisons were analyzed using ANOVA and Tukey-Kramer, and two-group comparisons were done using Welch’s *t*-test. *p* values <0.05 were considered significant. Significantly different taxa were identified using MaAsLin2. Multiple-testing correction was performed using the Benjamini-Hochberg procedure (FDR) to consider *p* < 0.05 and *q* ≤ 0.1. Sample size for each condition ranged from 3 to 5. Mice in different cages were treated identically and results were averaged within each group. Thus, our replicates are biological replicates.

## Supplementary information

Supplementary Information-New

Description of Additional Supplementary Data

Supplementary Data 1

Supplementary Data 2

Supplementary Data 3

Supplementary Data 4

Supplementary Data 5

Supplementary Data 6

Supplementary Data 7

Supplementary Data 8

Supplementary Data 9

Supplementary Data 10

Supplementary Data 11

Supplementary Data 12

## Data Availability

The metagenomics data have been deposited to Sequence Read Archive (SRA) of National Center for Biotechnology Information (NCBI) with a BioProject number: PRJNA870948. The source data for graphs and charts are provided in Supplementary Data 1–12.

## Code availability

No custom code was created. The codes used to analyze data are outlined above. Command scripts are available upon request. Specifically, codes used to analyze data using MaAsLin2 was obtained form https://huttenhower.sph.harvard.edu/maaslin/. Heatmaps were created using Heatmap() function in RStudio. PICRUSt2 code was obtained from https://github.com/picrust/picrust2.
