# Supplementary material for: Cftr deletion in mouse epithelial and immune cells differentially influence the intestinal microbiota
Source: Commun Biol. 2022 Oct 26;5:1130. doi: 10.1038/s42003-022-04101-5 (PMC9605958; doi:10.1038/s42003-022-04101-5)
Supplement: Supplementary file 1 — Supplementary Information-New [file 42003_2022_4101_MOESM1_ESM.pdf]

SUPPLEMENTARY INFORMATION

Cftr Deletion in Mouse Epithelial and Immune Cells Differentially Influence the Intestinal Microbiota

Callie E. Scull, Meng Luo, Scott Jennings, Christopher M. Taylor and Guoshun Wang

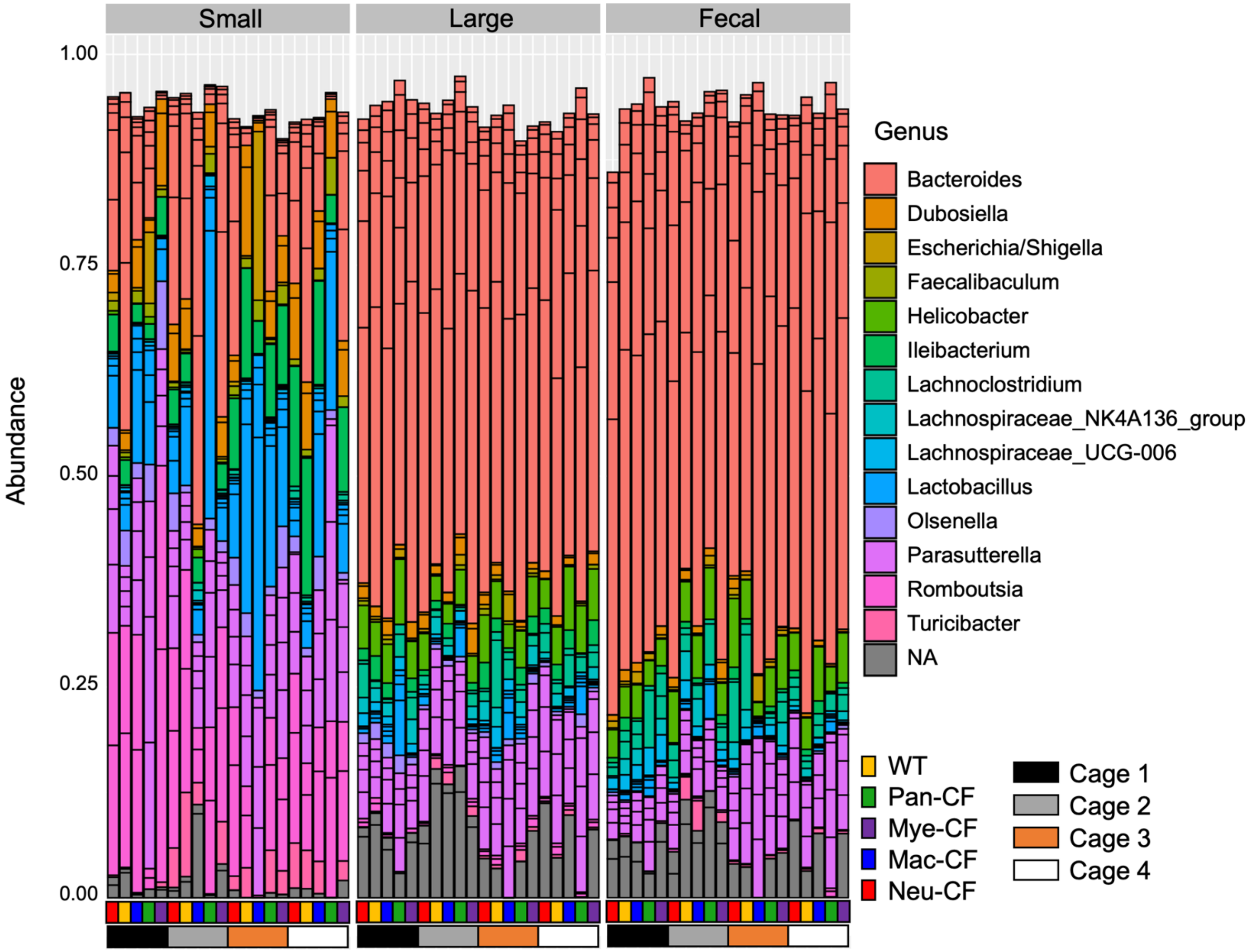

Supplementary Figure 1. Taxonomic bar graph for top 15 taxa of all samples from 3 locations of all genotypes of co-housed mice, which are sorted by cage.

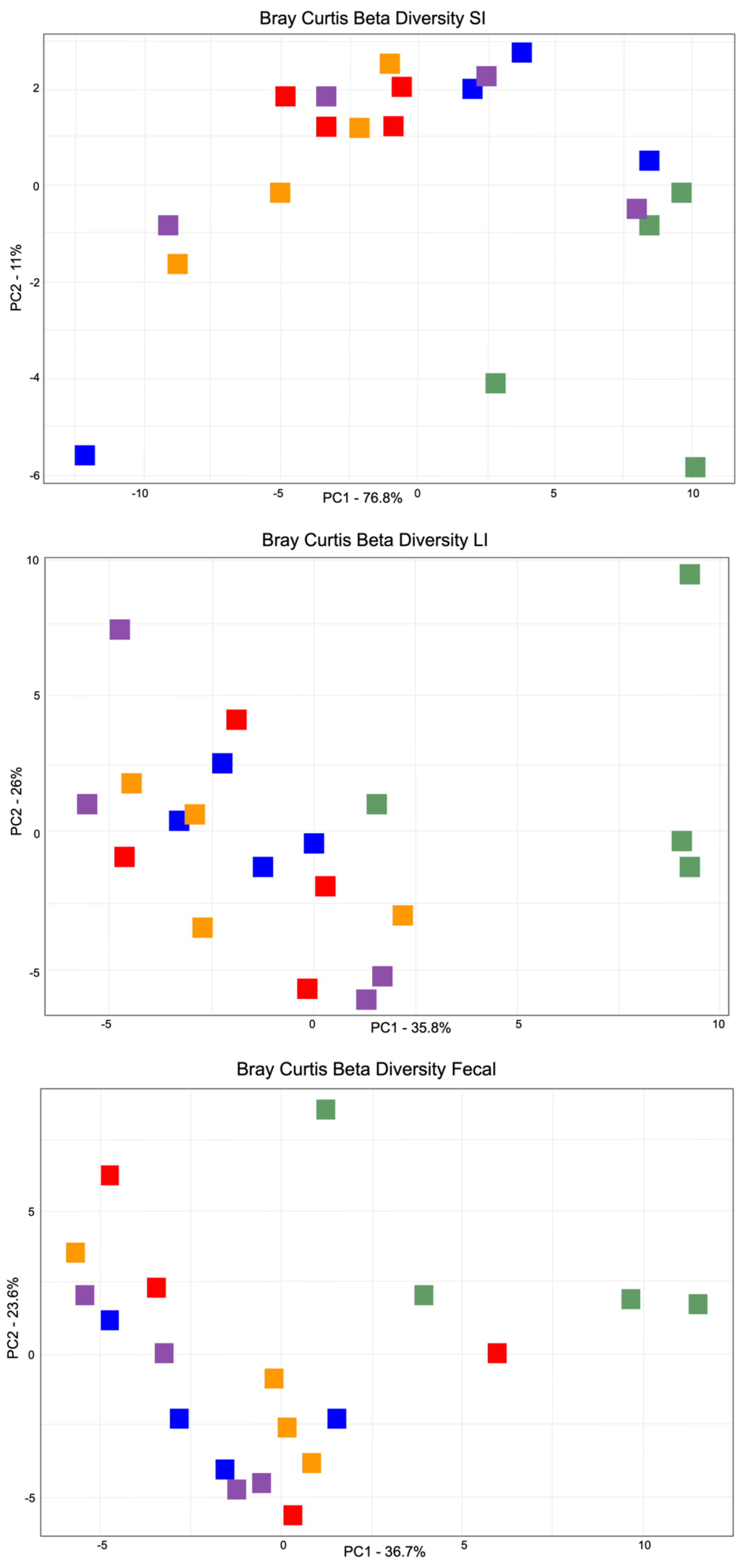

Supplementary Figure 2. Bray Curtis Dissimilarity PCA plots for co-housing mice by location (n=4 per genotype). SI: Small intestine; LI: Large intestine; WT ( ); Pan-CF ( ); Mye-CF ( ); Mac-CF ( ); Neu-CF ( ).

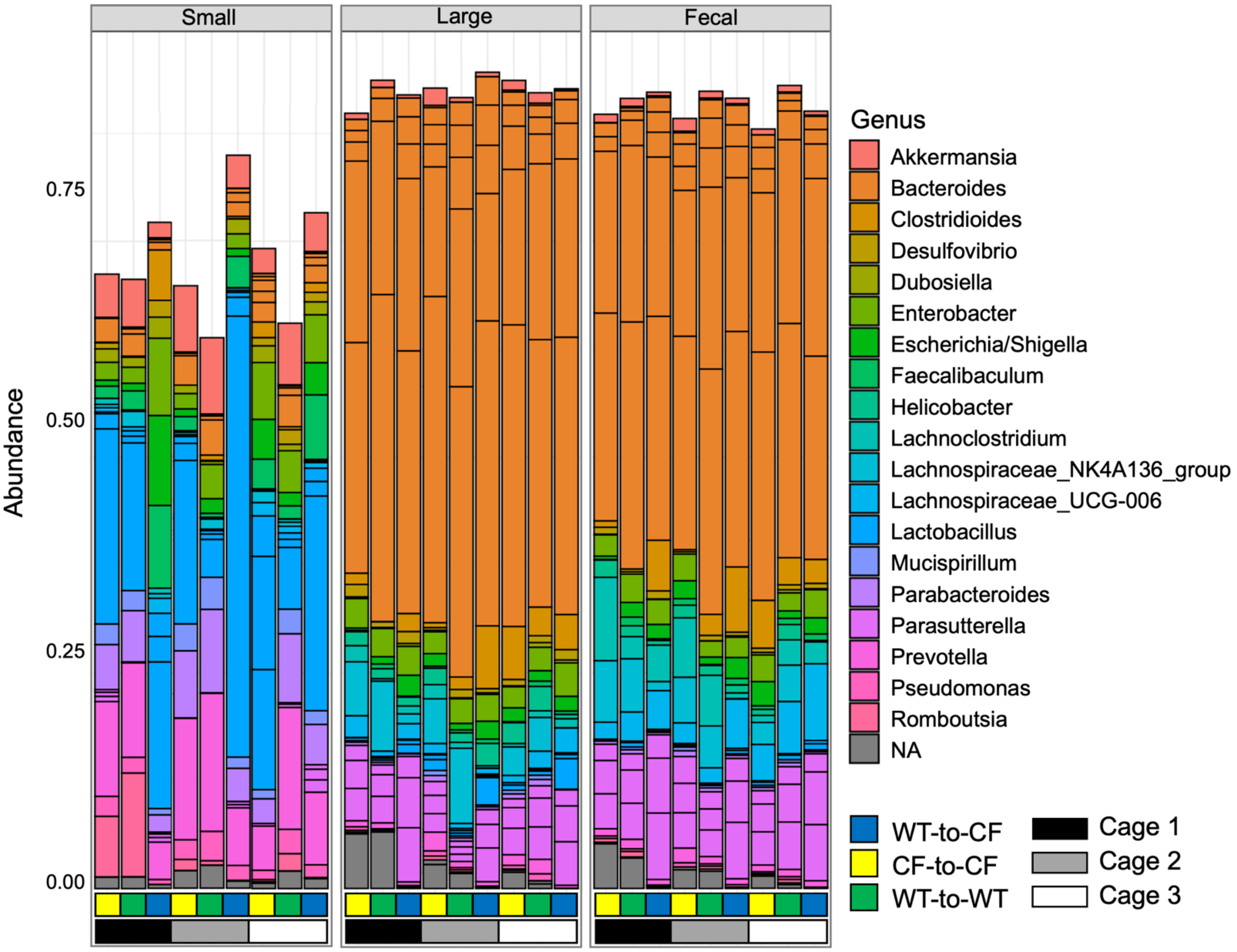

Supplementary Figure 3. Taxonomic bar graph for top 20 taxa of all samples from 3 locations of 3 BMT conditions, which are sorted by cage.

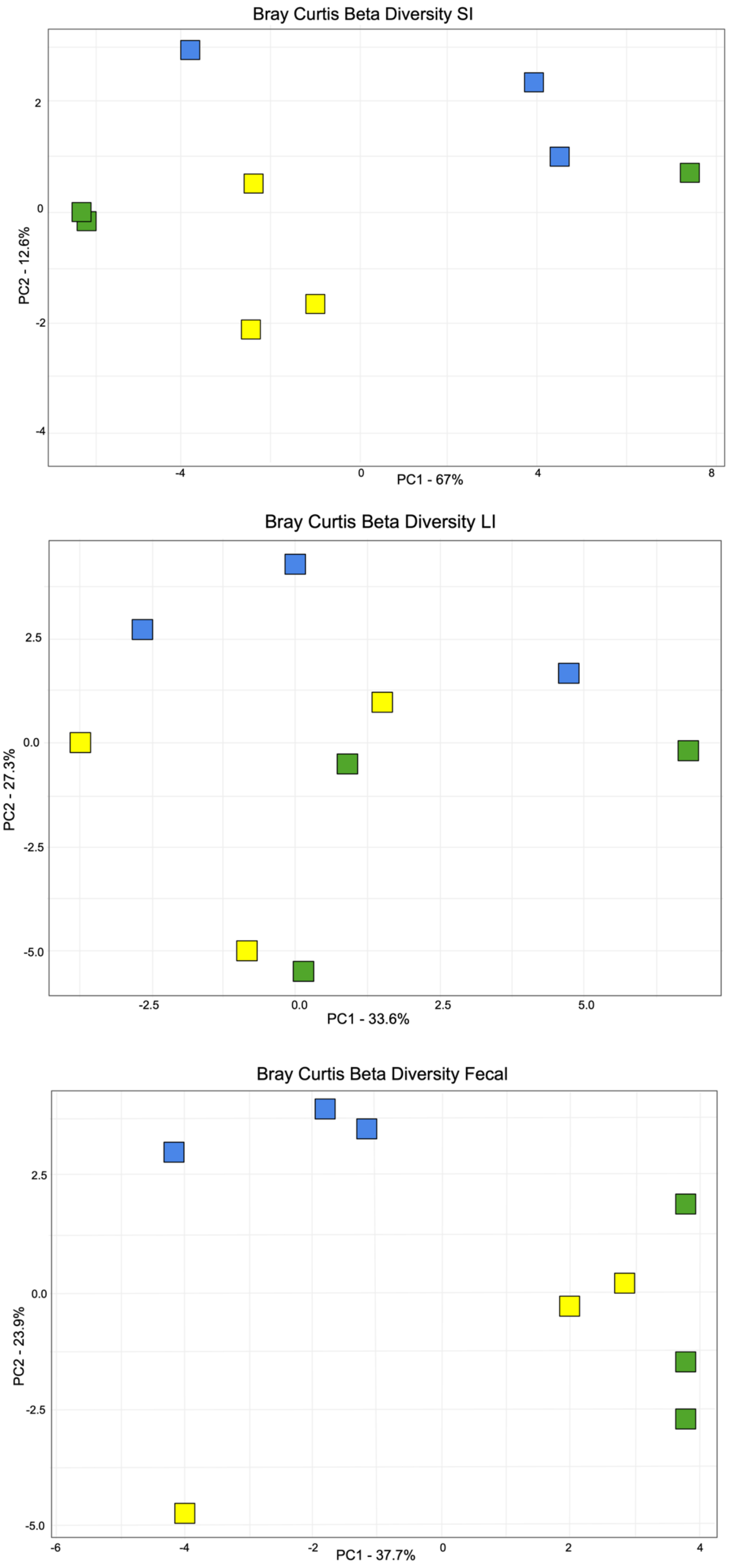

Supplementary Figure 4. PCA plots for BMT mice by location (n=3 per genotype). SI: Small intestine; LI: Large intestine. WT-to-WT (■); CF-to-CF (■); Blue - WT-to-CF (■).

Supplementary Table 1: Bacterial Taxa with Significant Difference in Relative Abundance (RA) (WT vs Pan-CF)

Small Intestine Taxa	Phylum	Class	WT RA (-log10)	Pan-CF RA (-log10)	P-Value	Q-Value
Kineothrix alysoides	Firmicutes	Clostridia	2.602059991	0	2.34E-05	0.002081
Desulfovibrio litoralis	Proteobacteria	Deltaproteobacteria	1.823908741	0	0.000607	0.027002
Bacteroides rodentium	Bacteroidetes	Bacteroidia	0.903089987	0	0.002212	0.028119
Turcibacter sanguinis	Firmicutes	Erysipelotrichia	1.301029996	0	0.002044	0.028119
Faecalibaculum rodentium	Firmicutes	Allobaculum	2.301029996	1.602059991	0.001626	0.028119
Desulfovibrio desulfuricans	Proteobacteria	Deltaproteobacteria	2.823908741	0	0.001982	0.028119
Ligilactobacillus faecis	Firmicutes	Bacilli	0	2.455931956	0.001782	0.028119
Clostridium scindens	Firmicutes	Clostridia	2.677780705	0	0.004273	0.042252
Breznakia pachnodae	Firmicutes	Erysipelotrichia	3.096910013	4	0.004146	0.042252
Parasutterella excrementihominis	Proteobacteria	Betaproteobacteria	1.602059991	1	0.005184	0.046136
Bacteroides caecimuris	Bacteroidetes	Bacteroidia	0.93930216	2.301029996	0.011099	0.089798

Large Intestine Taxa	Phylum	Class	WT RA (-log10)	Pan-CF RA (-log10)	P-Value	Q-Value
Kineothrix alysoides	Firmicutes	Clostridia	1.769551	0	9.45E-06	0.000832
Clostridium scindens	Firmicutes	Clostridia	2.346787	0	0.000356	0.015658
Lactobacillus gasseri	Firmicutes	Bacilli	0	1.455932	0.003335	0.043659
Ileibacterium valens	Firmicutes	Erysipelotrichia	1.69897	0	0.003158	0.043659
Ureaplasma parvum	Tenericutes	Mollicutes	2.939302	0	0.002807	0.043659
Ruminococcus gnavus	Firmicutes	Clostridia	2.481486	0	0.003203	0.043659
Anaerosacchariphilus polymeriproducens	Firmicutes	Clostridia	3.045757	0	0.003473	0.043659
Clostridium scindens	Firmicutes	Clostridia	2.522879	0	0.004305	0.047355
Romboutsia timonensis	Firmicutes	Clostridia	2.431798	3	0.009516	0.076773
Turcibacter sanguinis	Firmicutes	Erysipelotrichia	2.09691	0	0.009149	0.076773
Bifidobacterium pseudolongum	Actinobacteria	Actinomycetia	0	2.60206	0.009597	0.076773

Supplementary Table 2: Bacterial Taxa with Significant Difference in Relative Abundance (RA) (WT vs Mye-CF)

Small Intestine Taxa	Phylum	Class	WT RA (-log10)	Mye-CF RA (-log10)	P-Value	Q-Value
Desulfovibrio desulfuricans	Proteobacteria	Deltaproteobacteria	2.823908741	0	0.000321	0.028612

Supplementary Table 3: Bacterial Taxa with Significant Difference in Relative Abundance (RA) (WT vs Neu-CF)

Small Intestine Taxa	Phylum	Class	WT RA (-log10)	Neu-CF RA (-log10)	P-Value	Q-Value
Desulfovibrio piger	Proteobacteria	Deltaproteobacteria	2.823908741	0	0.000126	0.011299

Supplementary Table 4: Bacterial Taxa with Significant Difference in Relative Abundance (RA) (WT-to-CF vs. CF-to-CF)

Small Intestine Taxa	Phylum	Class	WT-to-CF RA (-log10)	CF-to-CF RA (-log10)	P-Value	Q-Value
Staphylococcus roterodami	Firmicutes	Bacilli	2.52287875	2.07058107	0.00195067	0.07537734
Bifidobacterium pseudolongum	Actinobacteria	Actinomycetia	2.1079054	0	0.00179579	0.07537734
Lactobacillus iners	Firmicutes	Bacilli	0	2.30103	0.00374149	0.07537734
Pseudoflavonifractor capillosus	Firmicutes	Clostridia	3.15490196	2.63827216	0.00313372	0.07537734
Bacteroides stercoris	Bacteroidetes	Bacteroidia	0	2.90308999	0.00171619	0.07537734
Blautia wexlerae	Firmicutes	Clostridia	0	2.75696195	0.00306683	0.07537734
Campylobacter gracilis	Proteobacteria	Epsilonproteobacteria	0	2.90308999	0.00212176	0.07537734
Oscillibacter ruminantium	Firmicutes	Clostridia	0	2.91009489	0.00384088	0.07537734

Large Intestine Taxa	Phylum	Class	WT-to-CF RA (-log10)	CF-to-CF RA (-log10)	P-Value	Q-Value
Lachnoclostridium pacaense	Firmicutes	Clostridia	0	1.22184875	0.00290354	0.12505822
Desulfovibrio litoralis	Proteobacteria	Deltaproteobacteria	0	1.7212464	0.00431962	0.12505822
Lactobacillus intestinalis	Firmicutes	Bacilli	2.60205999	0	0.00164471	0.12505822
Parabacteroides distasonis	Bacteroidetes	Bacteroidia	2.52287875	0	0.00500233	0.12505822
Clostridium scindens	Firmicutes	Clostridia	0	2.63827216	0.0063383	0.12676599

Supplementary Table 5: Bacterial Taxa with Significant Difference in Relative Abundance (RA) (WT-to-WT vs. WT-to-CF)

Small Intestine Taxa	Phylum	Class	WT-to-WT RA (-log10)	WT-to-CF RA (-log10)	P-Value	Q-Value
Bacteroides stercoris	Bacteroidetes	Bacteroidia	2.756962	0	0.00047365	0.07720471

Large Intestine Taxa	Phylum	Class	WT-to-WT RA (-log10)	WT-to-CF RA (-log10)	P-Value	Q-Value
Parabacteroides distasonis	Bacteroidetes	Bacteroidia	0	2.5228787	8.96E-05	0.00967339
Enterobacter cloacae	Proteobacteria	Gammaproteobacteria	0	2.2596373	0.00025004	0.01350206
Desulfovibrio litoralis	Proteobacteria	Deltaproteobacteria	1.552842	0	0.00088339	0.03180195
Lactobacillus iners	Firmicutes	Bacilli	2.9586073	0	0.00129285	0.03490704
Clostridium scindens	Firmicutes	Clostridia	2.4089354	0	0.00253777	0.05481588
Lachnoclostridium pacaense	Firmicutes	Clostridia	1.30103	0	0.0046442	0.08359568
