# Supplementary material for: Cftr deletion in mouse epithelial and immune cells differentially influence the intestinal microbiota
Source: Commun Biol. 2022 Oct 26;5:1130. doi: 10.1038/s42003-022-04101-5 (PMC9605958; doi:10.1038/s42003-022-04101-5)
Supplement: Supplementary file 2 — Description of Additional Supplementary Data [file 42003_2022_4101_MOESM2_ESM.docx]

Supplementary Data 1

Description: Source data for taxa graphs (Fig. 1b and Suppl. Fig. 1)

Supplementary Data 2

Description: Numerical data for OTU alpha graph (Fig. 2a)

Supplementary Data 3

Description: Numerical data for Shannon alpha graph (Fig. 2b)

Supplementary Data 4

Description: Numerical data for Faith’s PD alpha plot (Fig. 2c)

Supplementary Data 5

Description: Numerical data for Goblet cell graph (Fig. 3b)

Supplementary Data 6

Description: Source data for taxa graphs (Fig. 4b and Suppl. Fig. 3)

Supplementary Data 7

Description: Picrust2 numerical data for multi-genotype co-housing graph (Fig. 6)

Supplementary Data 8

Description: Picrust2 numerical data for BMT co-housing graph (Fig. 7).

Supplementary Data 9

Description: KEGG source data for significant features from Picrust2 analysis of multi-genotype co-housing experiment (Fig. 6).

Supplementary Data 10

Description: KEGG source data for significant features from Picrust2 analysis of BMT co-housing experiment (Fig. 7)

Supplementary Data 11

Description: OUT raw data from SILVA analysis for multi-genotype co-housing experiment.

Supplementary Data 12

Description: OTU raw data from SILVA analysis for BMT co-housing experiment.
