# Supplementary figures and images for: Cftr deletion in mouse epithelial and immune cells differentially influence the intestinal microbiota
Source: Commun Biol. 2022 Oct 26;5:1130. doi: 10.1038/s42003-022-04101-5 (PMC9605958; doi:10.1038/s42003-022-04101-5)

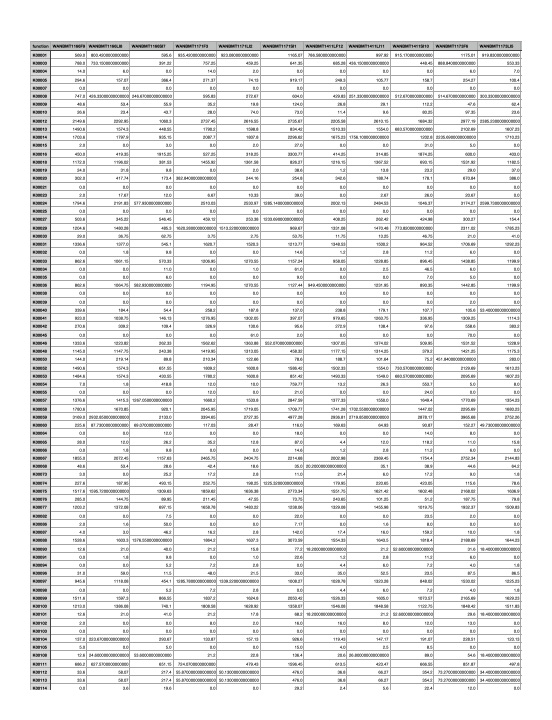

Supplement: Supplementary file 10 — Supplementary Data 8 [file 42003_2022_4101_MOESM10_ESM.zip › preview.jpg]

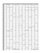

Supplement: Supplementary file 10 — Supplementary Data 8 [file 42003_2022_4101_MOESM10_ESM.zip › preview-micro.jpg]

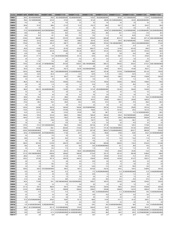

Supplement: Supplementary file 10 — Supplementary Data 8 [file 42003_2022_4101_MOESM10_ESM.zip › preview-web.jpg]
